# Analysis of Thermoelastic Interaction in a Polymeric Orthotropic Medium Using the Finite Element Method

**DOI:** 10.3390/polym14102112

**Published:** 2022-05-22

**Authors:** Ibrahim Abbas, Aatef Hobiny, Hashim Alshehri, Sorin Vlase, Marin Marin

**Affiliations:** 1 Mathematics Department, Faculty of Science, Sohag University, Sohag 82725, Egypt; ibrabbas7@science.sohag.edu.eg; 2 Nonlinear Analysis and Applied Mathematics Research Group (NAAM), Mathematics Department, King Abdulaziz University, Jeddah 22233, Saudi Arabia; ahobany@kau.edu.sa (A.H.); hmalshehri@kau.edu.sa (H.A.); 3 Department of Mechanical Engineering, Transilvania University of Brasov, 500036 Brasov, Romania; 4 Romanian Academy of Technical Sciences, 030167 Bucharest, Romania; 5 Department of Mathematics and Computer Science, Transilvania University of Brasov, 500036 Brasov, Romania

**Keywords:** thermal relaxation, governing equations, polymeric orthotropic material, finite element method

## Abstract

In this work, the finite element technique is employed to evaluate the effects of thermal relaxation durations on temperature, displacements, and stresses in a two-dimensional, polymeric, orthotropic, elastic medium. The problem is considered in a homogeneous, polymeric, orthotropic medium in the context of the Green and Lindsay model with two thermal relaxation times. The bounding surface of the half-space was subjected to a heat flux with an exponentially decaying pulse. Finite element techniques were used to solve the governing formulations, with eight-node isoparametric rectangular elements with three degrees of freedom (DOF) per node. The developed method was calculated using numerical results applied to the polymeric, orthotropic medium. The findings were implemented and visually shown. Finally, the results were displayed to demonstrate the differences between classical dynamic coupling (CT), the Lord–Shulman (LS) and the Green and Lindsay (GL) models.

## 1. Introduction

Over the last four decades, generalized thermoelastic models have drawn the significant interest of several researchers from the mathematical and technical perspective because of their remarkable, realistic implications in numerous regions which include continuum mechanics, nuclear engineering, aeronautics, high-energy particle accelerators, acoustics, etc. In materials science and solid mechanics, the polymeric, orthotropic medium has material properties varying along the three perpendicular axes, where each axis has double rotatory symmetry. Biot [1] constructed the coupled thermoelastic hypothesis to overcome the inconsistency which appeared when using the uncoupled hypothesis. The formulations of heat transfer and elasticity in this theorem are coupled. Several generalizations of the thermoelastic hypothesis were formulated by Lord and Shulman [2]. In 1980, the Lord–Shulman model was expanded upon by Dhaliwal and Sherief [3] to involve anisotropic cases. Lord and Shulman [2] presented the first generalized thermo-elastic model with one thermal relaxation time, whereas Green and Lindsay [4] obtained the second generalized thermo-elastic model in the case of two thermal relaxation times. Zenkour and Abbas [5] applied the finite element approach to investigate the effect of magnetic field in an infinite FG thermoelastic cylinder. Abo-Dahab and Abbas [6] investigated the effect of thermal relaxation times and the magnetic field with variable heat conduction in an infinite cylinder under thermal shock loading. Sarkar [7] discussed the wave propagations in elastic solids under magneto-thermoelastic theory using the time-fractional order two-temperature model. Lata and Kaur [8] studied the influences of inclined load and rotation on transversely isotropic material under thermal and magnetic fields. Alesemi [9] discussed the plane waves in a magneto-thermo-elastic anisotropic medium based upon the Lord and Shulman model. Singh [10] studied wave propagation in media with voids under the generalized thermoelastic model. Abbas and Abd-alla [11] investigated the effects of thermal relaxation on thermoelastic interaction in an infinite, orthotropic, elastic material with a cylindrical cavity. Khamis et al. [12] investigated the effects of ramp-type heating in a two-dimensional medium using the generalized thermo-visco-elastic model. Lata and Himanshi [13] studied the hall current in an orthotropic, magneto-thermoelastic solid with multi-dual-phase-lag models. Biswas [14] studied the thermal shock problem in porous, orthotropic material under the three-phase-lag theory. Biswas [15] used the eigenvalues approach to study the magneto-thermo-elastic problem in a transversely isotropic, hollow cylinder. Balubaid et al. [16] studied the dynamical behaviors of orthotropic, elastic materials using analytical solutions. Sarkar and Mondal [17] studied the thermoelastic plane wave under the modified Green–Lindsay theory with two-temperature formulations. The inclined load effect in an orthotropic, magneto-thermoelastic material with fractional-order heat transfer was explored by Lata and Himanshi [18]. Yadav [19] studied a magneto-thermoelastic wave in a rotating, orthotropic medium with diffusion. Lata and Himanshi [20] investigated the fractional influence of normal force in an orthotropic magneto-thermoelastic spinning solid of the GN-II type. Biswas [21] studied Rayleigh waves in a porous, orthotropic material with phase delays. Under the non-Fourier MGT thermoelastic model, Abouelregal et al. [22] investigated the thermo-visco-elastic behavior of an infinitely thin, orthotropic, hollow cylinder with changing characteristics. An orthotropic, magneto-thermoelastic solid was explored by Lata and Himanshi [23] using a multi-dual-phase-lag model and hall current. Several investigations have been carried out under the different theories in the recent literature [24,25,26,27,28,29,30,31,32,33,34,35,36,37,38,39]. In these papers, the authors used numerical and analytical approaches to solve several thermal and elastic wave problems.

In this work, the impacts of thermal relaxation times in a two-dimensional, polymeric, orthotropic medium upon the Green–Lindsay theory with two thermal relaxation times are studied. So, using the finite element method (FEM), the numerical solutions for the temperature increment, the displacement and the stress components are obtained. The results are plotted to show the difference between classical dynamic coupling (CT) in comparison with the Lord and Shulman (LS) and Green and Lindsay (GL) models.

## 2. Mathematical Model

For a polymeric, orthotropic, elastic, and homogeneous material, the basic formulations under the Green–Lindsay [4] model without body force and heating sources are introduced by the following equations.

The equations of motion can be given by:

(1) $$\sigma_{ij,j}=\rho \frac{\partial^{2}u_{i}}{\partial t^{2}},$$

The GL heat conduction equation can be written as:

(2) $$K_{i}T_{,ii}=\rho c_{e}\left(1+\tau_{1}\frac{\partial }{\partial t}\right)\frac{\partial T}{\partial t}+T_{o}\gamma_{i}\left(1+m\tau_{1}\frac{\partial }{\partial t}\right)u_{i,i},$$

The stress-displacement equations can be written as:

(3) $$\sigma_{ij}=c_{ijkl}e_{kl}-\gamma_{i}\left(T-T_{o}+\left(1+\tau_{2}\frac{\partial }{\partial t}\right)\right)\delta_{ij}.$$

We investigated an orthotropic and elastic, two-dimensional medium in this problem. The temperature and displacement components may be represented as follows:

(4) T=T(x,y,t), u=(u,v,0), u=u(x,y,t),v=v(x,y,t).

The equations of motion can be given by:

(5) $$c_{11}\frac{\partial^{2}u}{\partial x^{2}}+\left(c_{12}+c_{44}\right)\frac{\partial^{2}v}{\partial x\partial y}+c_{44}\frac{\partial^{2}u}{\partial y^{2}}-\gamma_{1}\left(1+\tau_{2}\frac{\partial }{\partial t}\right)\frac{\partial T}{\partial x}=\rho \frac{\partial^{2}u}{\partial t^{2}},$$

(6) $$c_{22}\frac{\partial^{2}v}{\partial y^{2}}+\left(c_{12}+c_{44}\right)\frac{\partial^{2}u}{\partial x\partial y}+c_{44}\frac{\partial^{2}v}{\partial x^{2}}-\gamma_{2}\left(1+\tau_{2}\frac{\partial }{\partial t}\right)\frac{\partial T}{\partial y}=\rho \frac{\partial^{2}v}{\partial t^{2}}.$$

The GL heat conduction equation can be written as:

(7) $$K_{1}\frac{\partial^{2}T}{\partial x^{2}}+K_{2}\frac{\partial^{2}T}{\partial y^{2}}=\rho c_{e}\left(1+\tau_{1}\frac{\partial }{\partial t}\right)\frac{\partial T}{\partial t}+T_{o}\left(1+m\tau_{1}\frac{\partial }{\partial t}\right)\left(\gamma_{1}\frac{\partial^{2}u}{\partial t\partial x}+\gamma_{2}\frac{\partial^{2}v}{\partial t\partial y}\right).$$

The stress-displacement equations can be written as:

(8) $$\sigma_{xx}=c_{11}\frac{\partial u}{\partial x}+c_{12}\frac{\partial v}{\partial y}-\gamma_{1}\left(1+\tau_{2}\frac{\partial }{\partial t}\right)T,\sigma_{yy}=c_{12}\frac{\partial u}{\partial x}+c_{22}\frac{\partial v}{\partial y}-\gamma_{2}\left(1+\tau_{2}\frac{\partial }{\partial t}\right)T,\;\sigma_{xy}=c_{44}\left(\frac{\partial u}{\partial y}+\frac{\partial v}{\partial x}\right),$$

where $\tau_{1},\tau_{2}$ are the thermal relaxation times; ρ is the density of the material; T is the increment of temperature; $c_{11}$, $c_{22}$, $c_{44},$ and $c_{12}$ are the elastic constants; $c_{e}$ is the specific heat; $\sigma_{xx},$ $\sigma_{yy},$ and $\sigma_{xy}$ are the stress components; t is the time; $T_{o}$ is the reference temperature; u and v are the displacement components; $K_{1}$ and $K_{2}$ are the thermal conductivity components; and $\gamma_{1}$ and $\gamma_{2}$ are the thermal stress coefficients. This model can be reduced to:

(i) (GL) refers to Green and Lindsay’s model
 $0<\tau_{1}<\tau_{2}\;,\;m=0$,
(ii) (LS) points to Lord and Shulman’s model
 $\tau_{1}>0,\;\tau_{2}=0,\;m=1$.
(iii) (CT) points to the classical, dynamically coupled model
 $\tau_{1}=\tau_{2}=m=0$,

## 3. Initial and Boundary Conditions

The initial conditions can be given by:

(9) $$T\left(x,y,0\right)=\frac{\partial T}{\partial t}=0,\;u=\frac{\partial u}{\partial t}=0,\;v\left(x,y,0\right)=\frac{\partial v}{\partial t}=0,\;t=0,$$

while the problem boundary conditions are presented by:

(10) $$-K_{1}\frac{\partial T\left(x,y,t\right)}{\partial x}=q_{o}\frac{t^{2}e^{-\frac{t}{\tau_{p}}}}{16\tau_{p}^{2}}H\left(a-\left|y\right|\right),u=0,\sigma_{xy}=0.0,$$

where $\tau_{p}$ is the characteristic time of pulse heat flux, $q_{o}$ is a constant, and H is the unit step function. To obtain the main fields in nondimensional forms, the non-dimensional parameters are taken:

(11) $$\left(x^{\prime },y^{\prime },u^{\prime },v^{\prime }\right)=\eta c\left(x,y,u,v\right),t^{\prime }=\eta c^{2}t,\;\left(\sigma_{xx}^{’},\sigma_{yy}^{’},\sigma_{xy}^{’}\right)=\frac{\left(\;\sigma_{xx},\;\sigma_{yy},\;\sigma_{xy}\right)}{c_{11}},T^{\prime }=\frac{T-T_{o}}{T_{o}},$$

where $\eta =\frac{\rho c_{e}}{K_{1}}$ and $c=\sqrt{\frac{c_{11}}{\rho }}$. By using the non-dimensional variables in Equation (11), the governing Equations (5)–(10) can be given (the dashes have been dropped for appropriateness):

(12) $$\frac{\partial^{2}u}{\partial x^{2}}+\left(s_{1}+s_{3}\right)\frac{\partial^{2}v}{\partial x\partial y}+s_{3}\frac{\partial^{2}u}{\partial y^{2}}-s_{4}\left(1+\tau_{2}\frac{\partial }{\partial t}\right)\frac{\partial T}{\partial x}=\frac{\partial^{2}u}{\partial t^{2}},$$

(13) $$s_{2}\frac{\partial^{2}v}{\partial y^{2}}+\left(s_{1}+s_{3}\right)\frac{\partial^{2}u}{\partial x\partial y}+s_{3}\frac{\partial^{2}v}{\partial x^{2}}-s_{5}\left(1+\tau_{2}\frac{\partial }{\partial t}\right)\frac{\partial T}{\partial y}=\frac{\partial^{2}v}{\partial t^{2}},$$

(14) $$\frac{\partial^{2}T}{\partial x^{2}}+s_{6}\frac{\partial^{2}T}{\partial y^{2}}=\left(1+\tau_{1}\frac{\partial }{\partial t}\right)\frac{\partial T}{\partial t}+\left(1+m\tau_{1}\frac{\partial }{\partial t}\right)\left(s_{7}\frac{\partial^{2}u}{\partial t\partial x}+s_{8}\frac{\partial^{2}v}{\partial t\partial y}\right),$$

(15) $$\sigma_{xx}=\frac{\partial u}{\partial x}+s_{1}\frac{\partial v}{\partial y}-s_{4}\left(1+\tau_{2}\frac{\partial }{\partial t}\right)T,\;\sigma_{yy}=s_{1}\frac{\partial u}{\partial x}+s_{2}\frac{\partial v}{\partial y}-s_{5}\left(1+\tau_{2}\frac{\partial }{\partial t}\right)T,\;\sigma_{xy}=s_{3}\left(\frac{\partial u}{\partial y}+\frac{\partial v}{\partial x}\right),$$

(16) $$u=0,\sigma_{xy}=0,\frac{\partial T\left(x,y,t\right)}{\partial x}=-q_{o}\frac{t^{2}e^{-\frac{t}{\tau_{p}}}}{16\tau_{p}^{2}}H\left(a-\left|y\right|\right),$$

where $s_{1}=\frac{c_{12}}{c_{11}}$, $s_{2}=\frac{c_{22}}{c_{11}}$, $s_{3}=\frac{c_{44}}{c_{11}}$, $s_{4}=\frac{T_{o}\gamma_{1}}{c_{11}}$, $s_{5}=\frac{T_{o}\gamma_{2}}{c_{11}}$, $s_{6}=\frac{K_{2}}{K_{1}}$, $s_{7}=\frac{\gamma_{1}}{\rho c_{e}}$, and $s_{8}=\frac{\gamma_{2}}{\rho c_{e}}$.

## 4. Finite Element Method

In this section, the basic formulations of homogeneous, polymeric, orthotropic material are summarized, followed by the corresponding finite element formulations. Abbas and his colleagues [40,41,42] presented the solutions for various problems under deference-generalized thermoelastic theories. The finite element formulation of thermoelastic diffusion can be obtained by using the standard procedure. In the finite element approach, the temperature change T and the displacement components u,v are related to the corresponding nodal values by

(17) $$T=\sum_{j=1}^{n}N_{j}T_{j}\left(t\right),u=\sum_{j=1}^{n}N_{j}u_{j}\left(t\right),v=\sum_{j=1}^{n}N_{j}v_{j}\left(t\right),$$

where $N_{j}$ are the shape functions, and m points to the number of nodes per element. The eight-node quadrilateral, the isoperimetric element, is used for the temperature and displacement computations. The shape functions and weighting functions coincide, hence:

(18) $$\delta T=\sum_{j=1}^{n}N_{j}\delta T_{j},\delta u=\sum_{j=1}^{n}N_{j}\delta u_{j},\delta v=\sum_{j=1}^{n}N_{j}\delta v_{j}.$$

In the absence of heat sources and body forces, the basic formulations are multiplied by the test functions and, after that, are integrated into the spatial domain Ω using the boundary Γ. The applications of integrations by parts and the use of the divergence theory decrease the order of the derivatives and allow the applications of the problem boundary conditions. Thus, the finite element formulations corresponding to the Formulations (12)–(14) can be introduced by

(19) $$\begin{array}{c} \int_{y_{1}}^{y_{2}}\int_{x_{1}}^{x_{2}}\frac{\partial \delta u}{\partial x}\left(\frac{\partial u}{\partial x}+s_{1}\frac{\partial v}{\partial y}-s_{4}\left(1+\tau_{2}\frac{\partial }{\partial t}\right)T\right)dxdy+\int_{x_{1}}^{x_{2}}\int_{y_{1}}^{y_{2}}\frac{\partial \delta u}{\partial y}\left(s_{3}\left(\frac{\partial u}{\partial y}+\frac{\partial v}{\partial x}\right)\right)dydx=\\ \int_{y_{1}}^{y_{2}}\delta u\left(\frac{\partial u}{\partial x}+s_{1}\frac{\partial v}{\partial y}-s_{4}\left(1+\tau_{2}\frac{\partial }{\partial t}\right)T\right)dy+\int_{x_{1}}^{x_{2}}\delta u\left(s_{3}\left(\frac{\partial u}{\partial y}+\frac{\partial v}{\partial x}\right)\right)dx, \end{array}$$

(20) $$\begin{array}{c} \int_{y_{1}}^{y_{2}}\int_{x_{1}}^{x_{2}}\frac{\partial \delta v}{\partial x}\left(s_{3}\left(\frac{\partial u}{\partial y}+\frac{\partial v}{\partial x}\right)\right)dxdy+\int_{x_{1}}^{x_{2}}\int_{y_{1}}^{y_{2}}\frac{\partial \delta v}{\partial y}\left(s_{1}\frac{\partial u}{\partial x}+s_{2}\frac{\partial v}{\partial y}-s_{5}\left(1+\tau_{2}\frac{\partial }{\partial t}\right)T\right)dydx=\\ \int_{y_{1}}^{y_{2}}\delta v\left(s_{3}\left(\frac{\partial u}{\partial y}+\frac{\partial v}{\partial x}\right)\right)dy+\int_{x_{1}}^{x_{2}}\delta v\left(s_{1}\frac{\partial u}{\partial x}+s_{2}\frac{\partial v}{\partial y}-s_{5}\left(1+\tau_{2}\frac{\partial }{\partial t}\right)T\right)dx, \end{array}$$

(21) $$\begin{array}{c} \int_{y_{1}}^{y_{2}}\int_{x_{1}}^{x_{2}}\frac{\partial \delta T}{\partial x}\frac{\partial T}{\partial x}dxdy+\int_{y_{1}}^{y_{2}}\int_{x_{1}}^{x_{2}}\partial T(\left(1+\tau_{1}\frac{\partial }{\partial t}\right)\frac{\partial T}{\partial t}+\left(1+m\tau_{1}\frac{\partial }{\partial t}\right)(s_{7}\frac{\partial^{2}u}{\partial t\partial x}+\\ s_{8}\frac{\partial^{2}v}{\partial t\partial y}))dxdy+s_{6}\int_{x_{1}}^{x_{2}}\int_{y_{1}}^{y_{2}}\frac{\partial \delta T}{\partial y}\frac{\partial T}{\partial y}dydx=\int_{y_{1}}^{y_{2}}\partial T\frac{\partial T}{\partial x}dy+s_{6}\int_{x_{1}}^{x_{2}}\partial T\frac{\partial T}{\partial y}dx. \end{array}$$

On the other hand, the temporal derivatives of the unknown variables must be determined by Newmark’s method of temporal integration or other methods (see Wriggers [43]).

## 5. Results and Discussion

For numerical calculations, the orthotropic material cobalt vide was chosen for the purposes of the numerical estimations. The constants value of this material can be given as in [44]:

$$T_{o}=298\left(k\right),\;a=0.5,\;\tau_{1}=0.05,\tau_{2}=0.08,\;t=0.5,\;c_{e}=427\;\left(J\right)\left({\mathrm{kg}}^{-1}\right)\left(k^{-1}\right),\;c_{12}=1.65\times 10^{11}\left(N\right)\left(m^{-2}\right),c_{11}=3.71\times 10^{11}\left(N\right)\left(m^{-2}\right),\;\rho =8836\left(\mathrm{kg}\right)\left(m^{-3}\right)\;c_{44}=1.51\times 10^{11}\left(N\right)\left(m^{-2}\right),c_{22}=3.581\times 10^{11}\left(N\right)\left(m^{-2}\right),\;K_{1}=100\;\left(W\right)\left(m^{-1}\right)\left(k^{-1}\right)\;K_{2}=25\;\left(W\right)\left(m^{-1}\right)\left(k^{-1}\right),\gamma_{1}=7.04\times 10^{6}\left(N\right)\left(k^{-1}\right)\left(m^{-2}\right),\gamma_{2}=6.9\times 10^{6}\;\left(N\right)\left(k^{-1}\right)\left(m^{-2}\right)$$

**Figure 1:**
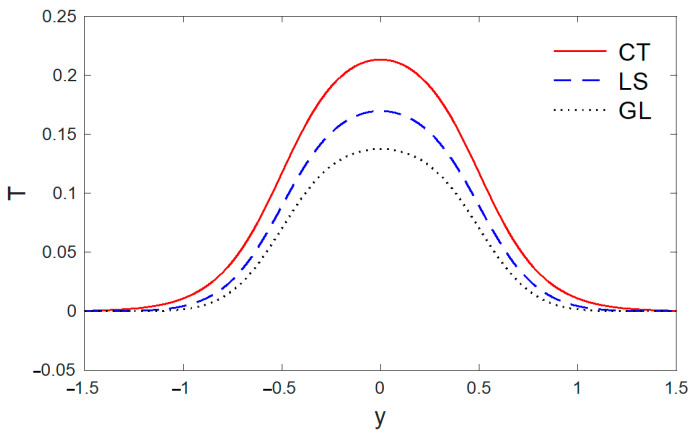
The variation of temperature T via y, with x=0.5 under the three models.

**Figure 2:**
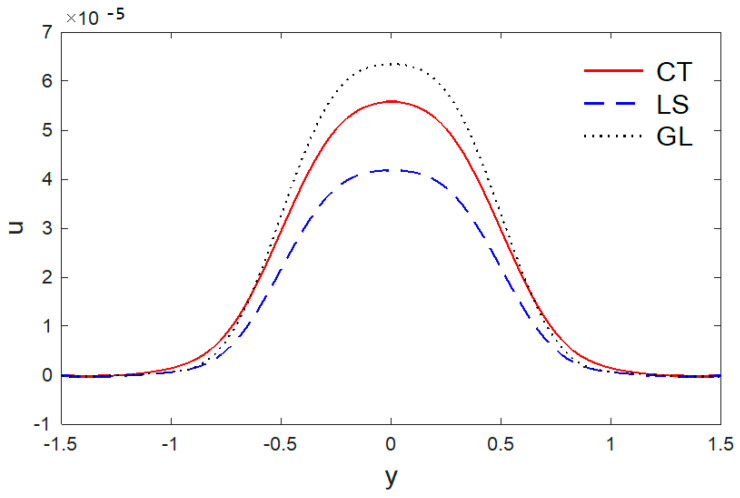
The horizontal displacement variations u via y, with x=0.5 under the three models.

**Figure 3:**
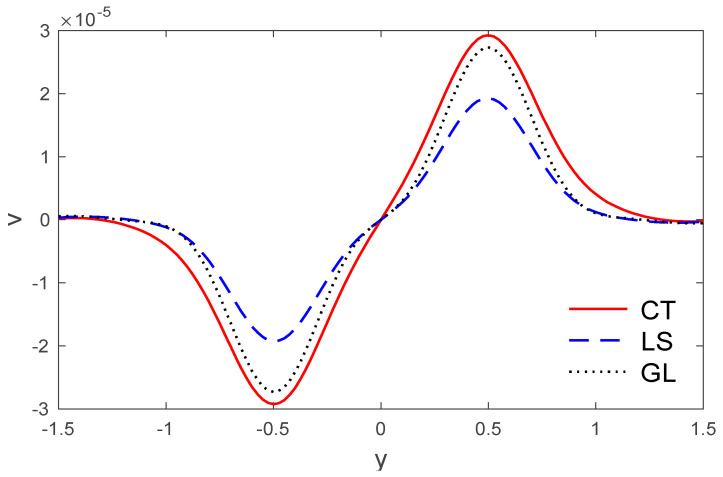
The variation of vertical displacement v via y, with x=0.5 under the three models.

**Figure 4:**
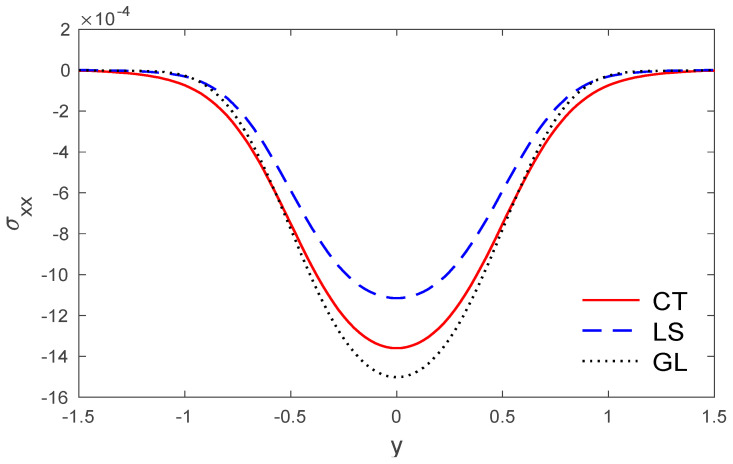
The stress variations $\sigma_{xx}$ via y, with x=0.5 under the three models.

**Figure 5:**
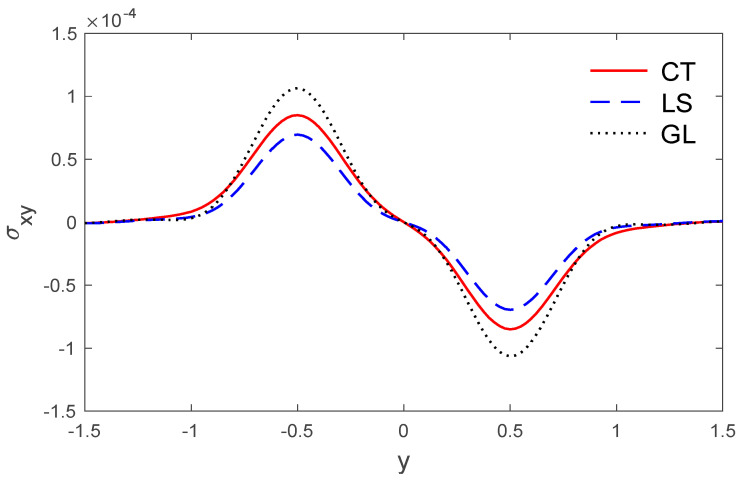
The stress variations $\sigma_{xy}$ via y, with x=0.5 under the three models.

**Figure 6:**
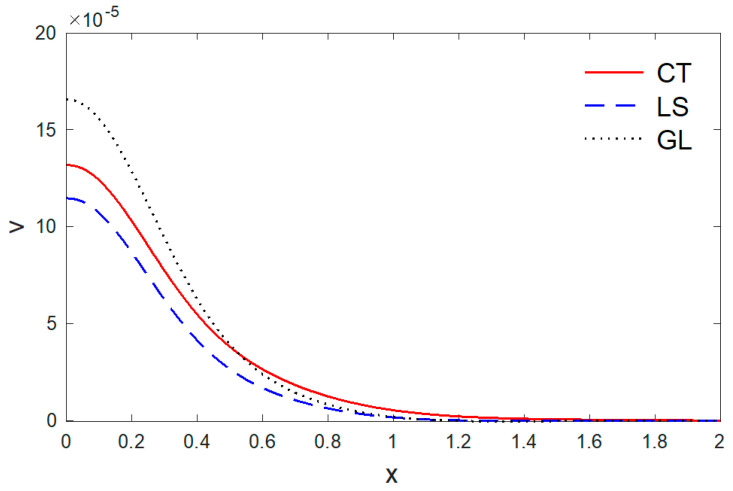
The vertical displacement variation v via x, with y =0.5 under the three models.

**Figure 7:**
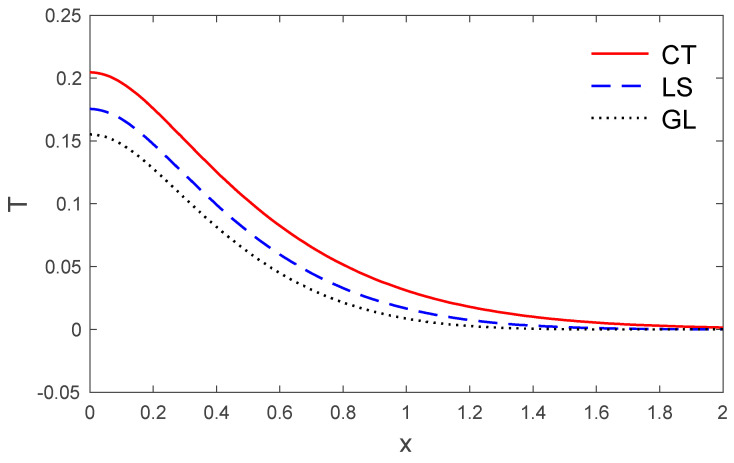
The temperature variation T via x, with y =0.5 under the three models.

**Figure 8:**
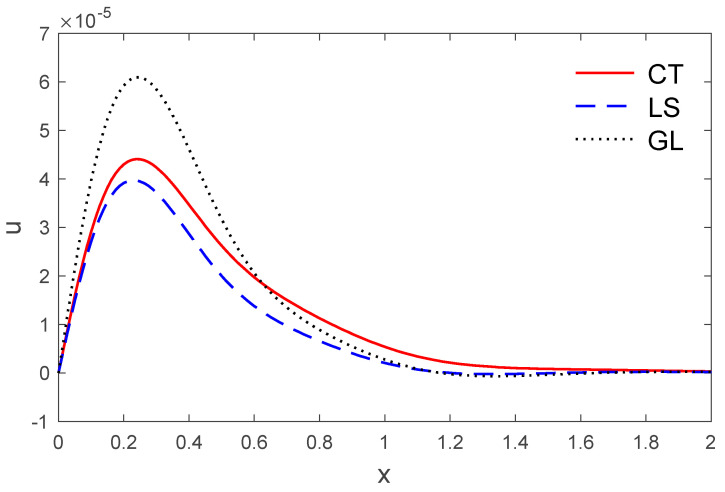
The horizontal displacement variation u via x, with y =0.5 under the three models.

**Figure 9:**
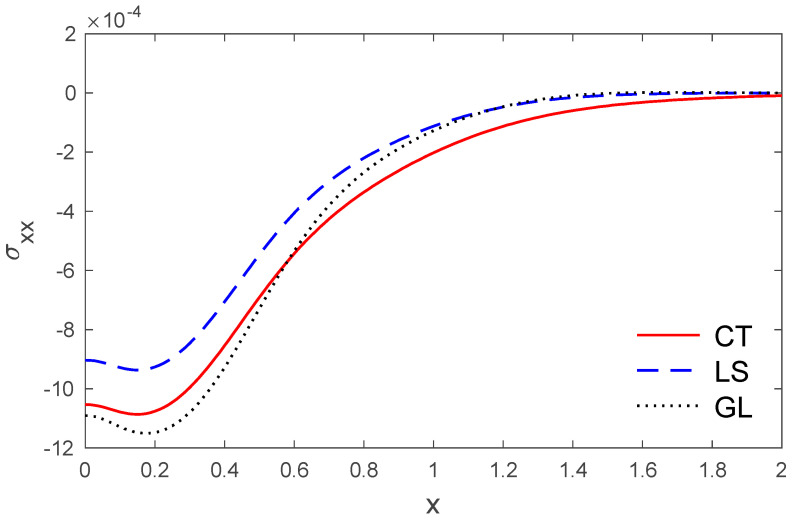
The stress variations $\sigma_{xx}$ via x with, y =0.5 under the three models.

**Figure 10:**
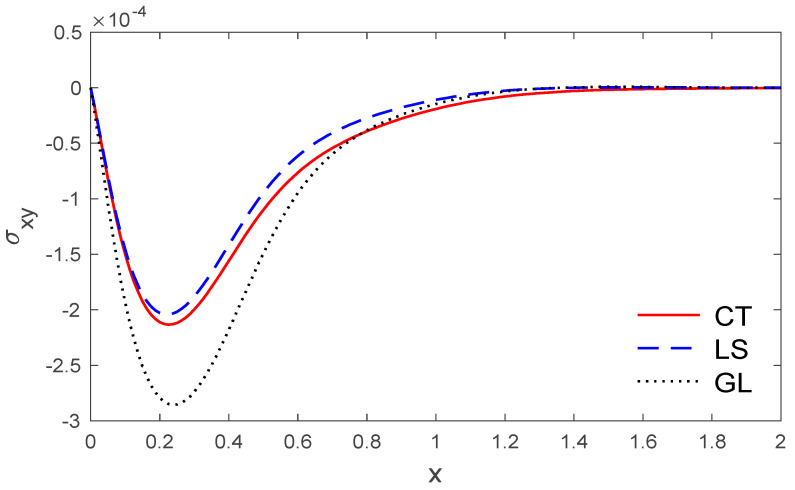
The variation of stress $\sigma_{xy}$ via x, with y =0.5 under the three models.

**Figure 11:**
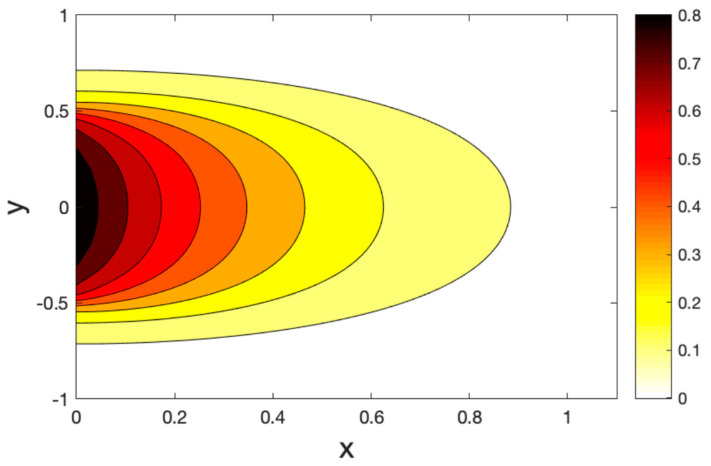
Contour plots of the temperature variations under the CT model.

**Figure 12:**
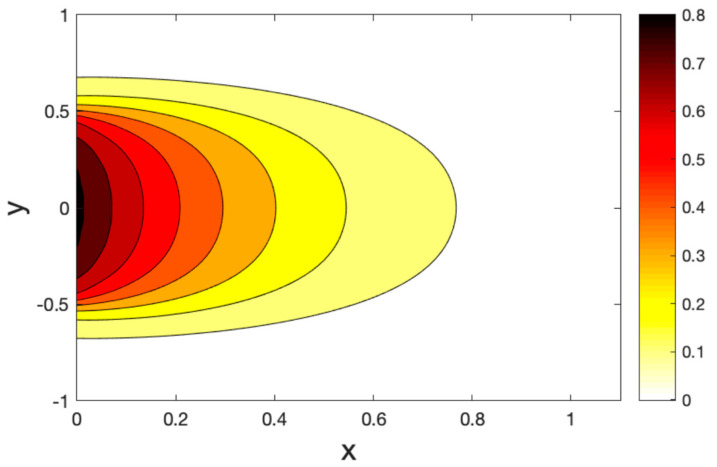
Contour plots of the temperature variations under the LS model.

**Figure 13:**
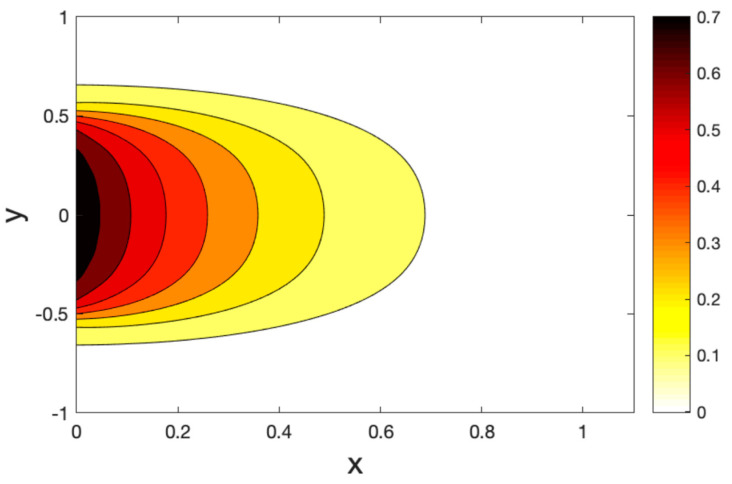
Contour plots of the temperature variations under the GL model.

The above data have been used to study the difference between the classical dynamic coupling (CT), Lord and Shulman (LS) and Green and Lindsay (GL) models in the distributions of temperature T, the components of displacement u, v, and the stress components $\sigma_{xx},\;\sigma_{xy}$. The medium is considered to be an orthotropic, elastic, two-dimensional material. The results are presented graphically with the distance 0<x<2 and −2<y<2 for three models, as in Figure 1, Figure 2, Figure 3, Figure 4, Figure 5, Figure 6, Figure 7, Figure 8, Figure 9, Figure 10, Figure 11, Figure 12 and Figure 13. Figure 1 displays the temperature variation via the distance y, and it points that the variations of temperature have maximum values at the length of the thermal surface (|y|≤0.5), and it starts to decrease just near the edges ((|y|≤0.5)), where the temperature regularly reduces and finally reaches a zero value. 

Figure 2 shows the horizontal displacement variations u via x. It should be noted that the horizontal displacement has a maximum value at the length of the heating surface (|y|≤0.5), and it begins to reduce just near the edge (y=±0.5), and then reduces to a zero value. 

The stress components $\sigma_{xx}$ and $\sigma_{xy}$ via y are presented in Figure 4 and Figure 5.

Figure 6, Figure 7, Figure 8, Figure 9 and Figure 10 depict the variations of the components of the temperature T, the displacements u, v, and the stresses $\sigma_{xx},\;\sigma_{xy}$ versus the distance x in the context of the three thermoelastic models when (t=0.5). It can be seen in Figure 6 that the values of temperature T reduce with the increase in distance x. At a larger distance from the boundary of the medium, the temperature field reaches closer and closer to zero, and, at last, becomes zero. However, a significant difference in the temperature field near the boundary plane is observed for all three models. The greater values of temperature are noticed for the classical dynamic coupling (CT) in comparison with the Lord and Shulman (LS) and Green and Lindsay (GL) models.

Figure 7 displays the variations of horizontal displacement u with respect to the distance x. It can be observed that the horizontal displacement u started from a zero value, which satisfies the problem’s boundary conditions. Figure 8 shows the variations of vertical displacement via x, which have a maximum value on the boundary and decrease with the increase of x. It can be observed that the vertical displacement reduces with the increase of the distance x.

Figure 9 and Figure 10 show the variations of stress components $\sigma_{xx}$ and $\sigma_{xy}$ with respect to the distance x. It can be observed that the stress magnitudes permanently start from maximum magnitudes for the stress $\sigma_{xx},$ while the stress component $\sigma_{xy}$ starts from a zero value that obeys the boundary condition.

Figure 11, Figure 12 and Figure 13 show the temperature field for three models of the hole contours. We found that the temperature changed in the restricted zones in a finite area, while the temperature did not change outside this area. In addition, there are areas with a temperature gradient much greater than that of another area. This means that the heat is carried out at a limited rate. As expected, it can be found that the thermal relaxation times $\tau_{1},\tau_{2}$ have the most significant impact on the values of all the fields studied.

## 6. Conclusions

This work has studied the effects of thermal relaxation times in a two-dimensional, polymeric, orthotropic medium. The resulting non-dimensional formulations were solved using the finite element method. The significant impacts of the thermal relaxation times are presented for the studying fields. Accordingly, we can consider the generalized thermoelasticity models with one and two thermal relaxation times as an advancement. The conclusions described in this work may be valuable for researchers working on low-temperature material science, mathematical physics, and thermodynamics, as well as the development of hyperbolic thermoelastic theories.

## Data Availability

Not applicable.

## References

[1] Biot M.A. (1956). Thermoelasticity and irreversible thermodynamics. J. Appl. Phys..

[2] Lord H.W., Shulman Y. (1967). A generalized dynamical theory of thermoelasticity. J. Mech. Phys. Solids.

[3] Dhaliwal R.S., Sherief H.H. (1980). Generalized thermoelasticity for anisotropic media. Q. Appl. Math..

[4] Green A., Lindsay K. (1972). Thermoelasticity. J. Elast..

[5] Zenkour A.M., Abbas I.A. (2014). Magneto-thermoelastic response of an infinite functionally graded cylinder using the finite element method. J. Vib. Control.

[6] Abo-Dahab S., Abbas I.A. (2011). LS model on thermal shock problem of generalized magneto-thermoelasticity for an infinitely long annular cylinder with variable thermal conductivity. Appl. Math. Model..

[7] Sarkar N. (2017). Wave propagation in an initially stressed elastic half-space solids under time-fractional order two-temperature magneto-thermoelasticity. Eur. Phys. J. Plus.

[8] Lata P., Kaur I. (2019). Effect of rotation and inclined load on transversely isotropic magneto thermoelastic solid. Struct. Eng. Mech..

[9] Alesemi M. Plane waves in magneto-thermoelastic anisotropic medium based on (L–S) theory under the effect of Coriolis and centrifugal forces. Proceedings of the International Conference on Materials Engineering and Applications.

[10] Singh B. (2007). Wave propagation in a generalized thermoelastic material with voids. Appl. Math. Comput..

[11] Abbas I.A., Abd-Alla A.E.N.N. (2008). Effects of thermal relaxations on thermoelastic interactions in an infinite orthotropic elastic medium with a cylindrical cavity. Arch. Appl. Mech..

[12] Khamis A., El-Bary A., Youssef H.M., Nasr A.M.A.A. (2018). A Two Dimensional Random Model in the Theory of Generalized Thermoviscoelasticty for a Thick Plate Subjected to Stochastic Ramp-Type Heating. J. Adv. Phys..

[13] Lata P., Himanshi (2021). Orthotropic magneto-thermoelastic solid with multi-dual-phase-lag model and hall current. Coupled Syst. Mech..

[14] Biswas S. (2020). Thermal shock problem in porous orthotropic medium with three-phase-lag model. Indian J. Phys..

[15] Biswas S. (2019). Eigenvalue approach to a magneto-thermoelastic problem in transversely isotropic hollow cylinder: Comparison of three theories. Waves Random Complex Media.

[16] Balubaid M., Abdo H., Ghandourah E., Mahmoud S. (2021). Dynamical behavior of the orthotropic elastic material using an analytical solution. Geomach. Eng..

[17] Sarkar N., Mondal S. (2020). Thermoelastic plane waves under the modified Green–Lindsay model with two-temperature formulation. ZAMM J. Appl. Math. Mech. Z. Angew. Math. Mech..

[18] Lata P., Himanshi H. (2022). Inclined load effect in an orthotropic magneto-thermoelastic solid with fractional order heat transfer. Struct. Eng. Mech..

[19] Yadav A. (2021). Magnetothermoelastic Waves in a Rotating Orthotropic Medium with Diffusion. J. Eng. Phys. Thermophys..

[20] Lata P., Himanshi H. (2022). Fractional effect in an orthotropic magneto-thermoelastic rotating solid of type GN-II due to normal force. Struct. Eng. Mech..

[21] Biswas S. (2021). Rayleigh waves in porous orthotropic medium with phase lags. Struct. Eng. Mech..

[22] Abouelregal A.E., Ahmad H., Badr S.K., Elmasry Y., Yao S.W. (2022). Thermo-viscoelastic behavior in an infinitely thin orthotropic hollow cylinder with variable properties under the non-Fourier MGT thermoelastic model. ZAMM-J. Appl. Math. Mech. Z. Angew. Math. Mech..

[23] Marin M., Abbas I., Vlase S., Craciun E.M. (2020). A Study of Deformations in a Thermoelastic Dipolar Body with Voids. Symmetry.

[24] Hobiny A., Alzahrani F., Abbas I., Marin M. (2020). The effect of fractional time derivative of bioheat model in skin tissue induced to laser irradiation. Symmetry.

[25] Marin M. (2010). Some estimates on vibrations in thermoelasticity of dipolar bodies. JVC/J. Vib. Control.

[26] Marin M. (1998). A temporally evolutionary equation in elasticity of micropolar bodies with voids. Bull. Ser. Appl. Math. Phys..

[27] Marin M., Othman M.I.A., Seadawy A.R., Carstea C. (2020). A domain of influence in the Moore–Gibson–Thompson theory of dipolar bodies. J. Taibah Univ. Sci..

[28] Hobiny A., Abbas I.A. (2018). Analytical solutions of photo-thermo-elastic waves in a non-homogenous semiconducting material. Results Phys..

[29] Hobiny A., Abbas I.A. (2021). A GN model of thermoelastic interaction in a 2D orthotropic material due to pulse heat flux. Struct. Eng. Mech..

[30] Hobiny A., Abbas I. (2021). Generalized thermoelastic interaction in a two-dimensional orthotropic material caused by a pulse heat flux. Waves Random Complex Media.

[31] Othman M.I.A., Said S., Marin M. (2019). A novel model of plane waves of two-temperature fiber-reinforced thermoelastic medium under the effect of gravity with three-phase-lag model. Int. J. Numer. Methods Heat Fluid Flow.

[32] Marin M., Othman M.I.A., Abbas I.A. (2015). An extension of the domain of influence theorem for generalized thermoelasticity of anisotropic material with voids. J. Comput. Theor. Nanosci..

[33] Marin M. (2010). Harmonic vibrations in thermoelasticity of microstretch materials. J. Vib. Acoust..

[34] Ebrahimi F., Nopour R., Dabbagh A. (2022). Effects of polymer’s viscoelastic properties and curved shape of the CNTs on the dynamic response of hybrid nanocomposite beams. Waves Random Complex Media.

[35] Ebrahimi F., Nopour R., Dabbagh A. (2021). Effect of viscoelastic properties of polymer and wavy shape of the CNTs on the vibrational behaviors of CNT/glass fiber/polymer plates. Eng. Comput..

[36] Ebrahimi F., Khosravi K., Dabbagh A. (2021). A novel spatial–temporal nonlocal strain gradient theorem for wave dispersion characteristics of FGM nanoplates. Waves Random Complex Media.

[37] Yarali E., Farajzadeh M.A., Noroozi R., Dabbagh A., Khoshgoftar M.J., Mirzaali M.J. (2020). Magnetorheological elastomer composites: Modeling and dynamic finite element analysis. Compos. Struct..

[38] Hobiny A.D., Abbas I.A. (2018). Theoretical analysis of thermal damages in skin tissue induced by intense moving heat source. Int. J. Heat Mass Transf..

[39] Abbas I.A., Alzahrani F.S., Elaiw A. (2018). A DPL model of photothermal interaction in a semiconductor material. Waves Random Complex Media.

[40] Abbas I.A., Youssef H.M. (2012). A Nonlinear Generalized Thermoelasticity Model of Temperature-Dependent Materials Using Finite Element Method. Int. J. Thermophys..

[41] Kumar R., Abbas I.A. (2013). Deformation due to thermal source in micropolar thermoelastic media with thermal and conductive temperatures. J. Comput. Theor. Nanosci..

[42] Dabbagh A., Rastgoo A., Ebrahimi F. (2019). Finite element vibration analysis of multi-scale hybrid nanocomposite beams via a refined beam theory. Thin-Walled Struct..

[43] Wriggers P. (2008). Nonlinear Finite Element Methods.

[44] Singh B., Pal S. (2020). Magneto-thermoelastic interaction with memory response due to laser pulse under Green-Naghdi theory in an orthotropic medium. Mech. Based Des. Struct. Mach..

